# Clinical relevance of gene mutations and rearrangements in advanced differentiated thyroid cancer

**DOI:** 10.1016/j.esmoop.2023.102039

**Published:** 2023-10-23

**Authors:** M. Nannini, A. Repaci, M.C. Nigro, A. Colapinto, V. Vicennati, T. Maloberti, E. Gruppioni, A. Altimari, E. Solaroli, E. Lodi Rizzini, F. Monari, A. De Leo, S. Damiani, U. Pagotto, M.A. Pantaleo, D. de Biase, G. Tallini

**Affiliations:** 1 Department of Medical and Surgical Sciences (DIMEC), University of Bologna, Bologna; 2 Medical Oncology, IRCCS Azienda Ospedaliero-Universitaria di Bologna, Bologna; 3 Division of Endocrinology and Diabetes Prevention and Care, IRCCS Azienda Ospedaliero-Universitaria di Bologna, Bologna; 4 Solid Tumor Molecular Pathology Laboratory, IRCCS Azienda Ospedaliero-Universitaria di Bologna, Bologna; 5 Endocrinology Unit-Azienda USL di Bologna, Bologna; 6 Division of Radiation Oncology, IRCCS Azienda Ospedaliero-Universitaria di Bologna, Sant’Orsola-Malpighi Hospital, Bologna; 7 Pathology Unit, Department of Pathology, Bellaria & Maggiore Hospital, AUSL di Bologna, Bologna; 8 Department of Pharmacy and Biotechnology (FaBiT), University of Bologna, Bologna, Italy

**Keywords:** advanced differentiated thyroid cancer, radioactive iodide refractory, *TERT* promoter mutations, *TP53* mutations, NTRK fusions, RET fusions

## Abstract

**Background:**

Tumor genotyping is becoming crucial to optimize the clinical management of patients with advanced differentiated thyroid cancer (DTC); however, its implementation in clinical practice remains undefined. We herein report our single-center experience on molecular advanced DTC testing by next-generation sequencing approach, to better define how and when tumor genotyping can assist clinical decision making.

**Materials and methods:**

We retrospectively collected data on all adult patients with advanced DTC who received molecular profiling at the IRCSS Sant’Orsola-Malpighi Hospital from 2008 to 2022. The genetic alterations were correlated with radioactive iodide refractory (RAI-R), RAI uptake/disease status, and time to RAI resistance (TTRR) development.

**Results:**

A significant correlation was found between RAI-R development and genetic alterations (*P* = 0.0001). About 48.7% of RAI-R cases were positive for *TERT/TP53* mutations (as both a single event and comutations with other driver gene alterations, such as *BRAF* mutations, *RAS* mutations, or gene fusions), while the great majority of RAI-sensitive cases carried gene fusions (41.9%) or were wild type (WT; 41.9%). RAI uptake/disease status and time to TTRR were significantly associated with genetic alterations (*P* = 0.0001). In particular, DTC with *TERT/TP53* mutations as a single event or as comutations displayed a shorter median TTRR of 35.4 months (range 15.0-55.8 months), in comparison to the other molecular subgroups. *TERT/TP53* mutations as a single event or as comutations remained independently associated with RAI-R after Cox multivariate analysis (hazard ratio 4.14, 95% CI 1.51-11.32; *P* = 0.006).

**Conclusions:**

Routine testing for genetic alterations should be included as part of the clinical workup, for identifying both the subset of more aggressive tumors and the subset of tumors harboring actionable gene fusions, thus ensuring the appropriate management for all patients with advanced DTC.

## Highlights

• RAI resistance and time to RAI-R development in advanced DTC are significantly correlated with genetic profiling.
• Genetic testing allows for identifying the subset of more aggressive advanced DTC and those with actionable gene fusions.
• Genetic testing should be included as part of the clinical workup and carried out as early as possible in all advanced DTCs.

## Introduction

In the past years, the biological background of differentiated thyroid cancer (DTC) has been deeply investigated, resulting in the identification of molecular alterations that show remarkable genotype–histologic phenotype correlation with relevant impact on clinical practice. As widely known, the large majority of DTCs are characterized by mutually exclusive driver events, either point mutations or gene rearrangements, involved in the mitogen-activated protein kinase (MAPK) pathway and the phosphatidylinositol-3 kinase (PI3K)/protein kinase B (AKT) pathway.^1^, ^2^, ^3^ In particular, nonoverlapping mutations of *BRAF* (60%), *NRAS* (4%), *HRAS* (1.5%), and *KRAS* (0.3%) genes are found in conventional papillary thyroid carcinoma (PTC)—that is, those PTCs that grow making papillae—including classical and tall cell subtypes.^1^, ^2^, ^3^

Gene rearrangements—involving *RET* (8%), *BRAF* (2%), *NTRK1/3* (2%), *ALK* (1%), *FGFR2* (0.3%), and *LTK* (0.3%)—occur in a smaller subset of conventional PTCs. Conversely, mutations in *H/K/NRAS* (40%), and more rarely in *DICER1* (7%), *EIF1AX* (5%), *EZH1* (7%), *SPOP* (3%), *IDH1* (1%), and *SOS1* (1%), together with *PAX8/PPARG* rearrangements are found in follicular patterned carcinomas such as follicular thyroid carcinoma (FTC) and encapsulated follicular variant papillary carcinoma.^1^, ^2^, ^3^, ^4^, ^5^, ^6^ *TERT* promoter mutations—occurring in ∼12% and 18% of PTCs and FTCs, respectively—have been demonstrated to have negative prognostic implications in many studies.^7^, ^8^, ^9^, ^10^, ^11^

These advances have led to a shift in treatment paradigms for radioactive iodide refractory (RAI-R) DTC, for which multityrosine kinase inhibitors are becoming the standard of care.^12^, ^13^, ^14^

Therapeutic options for RAI-R DTC have further expanded with the recent advent of NTRK and RET inhibitors, which have shown marked and durable responses in patients with metastatic/advanced RAI-R DTC positive for *NTRK* or *RET* gene fusions.^12^^,^^15^, ^16^, ^17^, ^18^, ^19^

Without a doubt, in this scenario, tumor genotyping is becoming crucial to optimize the clinical management of patients with advanced DTC. However, its implementation into the clinical practice is still debated and detailed recommendations on timing and algorithm to use are still undefined. We herein report our single-center experience on molecular testing of patients with advanced DTC with the aim of better defining how and when tumor genotyping can assist clinical decision making.

## Material and methods

### Patients

In this retrospective, single-center, observational study, we collected data on all adult patients with DTC referred to the IRCSS Sant’Orsola-Malpighi Hospital from 2008 to 2022, for whom molecular testing by next-generation sequencing (NGS) was carried out during the disease course for clinical reasons, including distant metastases at presentation, disease recurrence, or progressive RAI-R. All cases were histologically reviewed and only confirmed cases of DTC have been included. All pathological, clinical, and follow-up data were anonymously collected from inpatient and outpatient medical records in an electronic database. Confirmed written consent for molecular testing was obtained from all patients.

### Molecular analysis

Gene mutations and gene fusions were evaluated on DNA and RNA obtained from cytologic or histologic samples. Nucleic acids were extracted from two to four 10-μm-thick formalin-fixed paraffin-embedded tissue sections: areas with the highest tumor cell enrichment were selected after examination of hematoxylin and eosin control-stained slides. For cytology analysis, nucleic acids were extracted from cells scraped directly from the stained smears, using only those with the highest tumor cell enrichment and best cytologic preservation of lesional cells. DNA analysis was carried out using a laboratory-developed multigene panel able to analyze the regions of 28 genes (human reference sequence hg19/GRCh37, total of 330 amplicons).^20^ RNA analysis for gene fusions was carried out using the Oncomine Focus Assay Panel (Thermo Fisher Scientific, Waltham, MA). Amplicon libraries were sequenced with a Gene Studio S5 Prime sequencer (Thermo Fisher Scientific) as previously described.^21^ Only nucleotide variations detected in both strands and at least 5% of the total number of reads analyzed were considered for the mutational calls.^21^ Ion Reporter Software (version 5.18; Thermo Fisher Scientific) and Integrative Genomics Viewer 2.12.2 tool (Available at http://software.broadinstitute.org/software/igv/, accessed on February 2023) were used to analyze the obtained sequences. The VarSome database (https://varsome.com/, accessed on February 2023) was used to evaluate the pathogenicity of each mutation.

### Statistical analysis

Comparisons between main clinicopathological features (sex, media age, histotypes, T-stage, N-stage, and multifocality) and molecular subgroups [*BRAF* mutations, *RAS* mutations, *TERT/TP53* mutations as a single event, gene fusions, and wild type (WT)] were carried out with the Pearson’s chi-square and analysis of variance tests. Likewise, comparisons between M-stage, RAI-R development, and RAI uptake/disease status and molecular subgroups (*BRAF* mutations only, *RAS* mutations only, *TERT/TP53* mutations as a single event or as comutations with other driver gene alterations, gene fusions, and WT) were carried out with Pearson’s chi-square. Time to RAI resistance (TTRR) was measured between the date of diagnosis and the date of the first evidence of loss RAI uptake, censoring patients who were alive without RAI-R on the date of the last follow-up. Analysis of TTRR among molecular subgroups (*BRAF* mutations only, *RAS* mutations only, *TERT/TP53* mutations as a single event or as comutations with other driver gene alterations, gene fusions, and WT) was carried out using the Kaplan–Meier method and log-rank test. To identify independent predictive factors for RAI-R, univariate and multivariate Cox proportional hazard regression models were carried out. All tests were carried out with a significance level of *P* < 0.05. Statistical analyses were carried out using IBM SPSS Statistics for Windows Version 19.0 (IBM Corporation, Armonk, NY).

## Results

### Patients’ characteristics and genetic alterations

A total of 82 consecutive adult patients affected by metastatic/advanced DTC have been retrospectively included. The main patients’ characteristics are listed in Supplementary Table S1, available at https://doi.org/10.1016/j.esmoop.2023.102039. The median age at diagnosis was 47 years (range 20-86 years) and 65.9% were female. Tumor histology was PTC in 73.2%, including infiltrative follicular variant PTC—tumors with BRAF p.V600E-like molecular profile according to current classification,^22^ encapsulated follicular variant PTC in 7.3%, FTC in 13.4%, and oncocytic thyroid carcinoma of follicular cells (OCC) in 6.1%.

Presentation was multifocal within the thyroid gland in 53.7% of cases and 19.5% of cases had distant metastases at the time of diagnosis.

Molecular analysis was carried out from the primary tumor, lymph node metastases, and distant metastases in 86.6%, 9.8%, and 3.7%, respectively. In 89% of cases, histologic specimens and in 11% cytological specimens were analyzed. In 33 cases (40.2%), at least one DNA mutation was identified, and 25 cases (30.5%) were positive for gene fusions, of which there was one *RET-CCDC6*-positive case in association with *TERT* promoter + *PIK3CA* + *TP53* comutations. All the remaining 24 cases (29.3%) were WT (Supplementary Table S2, available at https://doi.org/10.1016/j.esmoop.2023.102039).

**Figure 1:**
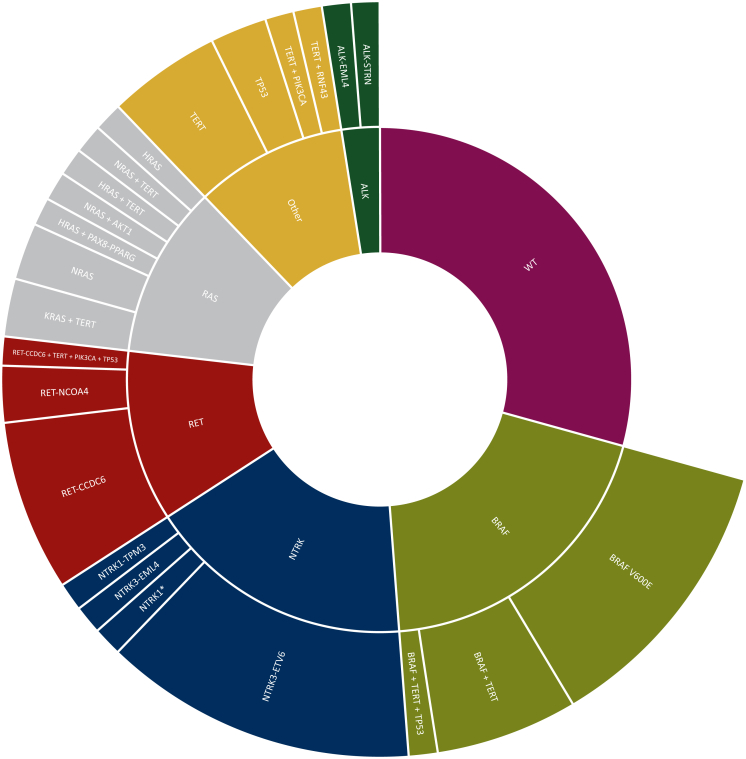
The spectrum of genetic alterations identified in 82 patients with metastatic/advanced differentiated thyroid cancer: in 33 cases (40.2%) at least one DNA mutation was identified, 25 cases (30.5%) were positive for gene fusions, and the remaining 24 cases (29.3%) were wild type (WT). ∗Gene fusion identified by FISH, undefined rearrangement partner.

Among the mutated subgroup, *BRAF* p.V600E mutation was found in 16 cases (19.5%), while *RAS* variants (*NRAS*, *n =* 4; *HRAS*, *n =* 3; and *KRAS*, *n =* 2) were found in 9 cases (11%). *BRAF* p.V600E and *RAS* mutations were single events in 10 and 3 cases, respectively. In one case, *HRAS* mutation occurred in association with *PAX8-PPARG* fusion. Substitutions in *TERT* promoter were found in 17 cases (20.7%) and in 11 tumors these occurred as comutations. Other less common mutations were *TP53* (4 cases, 4.9%, of which 2 cases were single events), *PIK3CA* (1 comutated case, 1.2%), *AKT1* (1 comutated case, 1.2%), and *RNF43* (1 comutated case, 1.2%). In the gene fusion-positive subgroup, *NTRK1* and *NTRK3* rearrangements were the most common, found in 14 cases (17.1%), followed by *RET* and *ALK* rearrangements, which were found in 9 (11.0%) and 2 cases (2.4%), respectively (Figure 1). In one case, *RET* fusion was found in association with *PIK3CA*, *TP53,* and *TERT* comutations (Supplementary Figure S1, available at https://doi.org/10.1016/j.esmoop.2023.102039).

### Clinicopathological features according to genetic alterations

First, cases have been divided into three main subgroups: mutation-positive DTC, fusion-positive DTC, and WT-DTC. The mutation-positive DTC and fusion-positive DTC cases have also been stratified by the type of molecular event (*BRAF* mutations, *RAS* mutations, *TERT/TP53* mutations as single-event, gene fusions). The main clinicopathological features of each molecular subgroup are listed in Table 1.

**Table 1:** Clinicopathological characteristics according to genetic alterations

	*BRAF*	*RAS*	*TERT/TP53* (single event)	Gene fusions	Wild type	*P* value
**Age, years**	44 (22-79)	50 (30-69)	70 (54-86)	41 (20-84)	48 (24-75)	**0.0001**
**Sex**						
F	10 (62.5)	4 (44.4)	5 (62.5)	18 (72.0)	17 (70.8)	ns
M	6 (37.5)	5 (55.6)	3 (37.5)	7 (28.0)	7 (29.2)	
**Histotype**						
PTC	16 (100)	1 (11.1)	4 (50)	25 (100)	14 (58.3)	**0.0001**
E-FV-PTC	—	4 (66.7)	—	—	2 (33.3)	
FTC	—	7 (77.8)	3 (37.5)	—	7 (29.2)	
OCC	—	1 (11.1)	1 (12.5)	—	3 (12.5)	
**T-stage**						
T1	6 (37.5)	3 (33.3)	—	9 (36.0)	8 (33.3)	ns
T2	2 (12.5)	—	1 (12.5)	5 (20.0)	8 (33.3)	
T3	6 (37.5)	4 (44.4)	3 (37.5)	8 (32.0)	8 (33.3)	
T4	1 (6.3)	2 (22.2)	2 (25.0)	2 (8.0)	—	
NE	1 (6.3)	—	2 (25.0)	—	—	
**N-stage**						
N0/Nx	4 (25.0)	8 (88.9)	3 (37.5)	7 (28.0)	20 (83.3)	**0.0001**
N1a	5 (31.3)	1 (11.1)	2 (25.0)	6 (24.0)	—	
N1b	6 (37.5)	—	1 (12.5)	11 (44.0)	4 (16.7)	
NE	1 (6.3)	—	2 (25.0)	1 (84.0)	—	
**Multifocality**						
Yes	11 (68.8)	2 (22.2)	—	13 (52.0)	8 (33.3)	ns
NE	1 (6.3)	—	2 (25.0)	1 (4.0)	—	

E-FV-PTC, encapsulated follicular variant papillary thyroid carcinoma; FTC, follicular thyroid carcinoma; NE, not evaluable (no primary surgery); ns, not significant; OCC, oncocytic thyroid carcinoma of follicular cells; PTC, papillary thyroid carcinoma.

Bold indicates the comparisons between main clinicopathological features (sex, media age, histotypes, T-stage, N-stage, and multifocality) and molecular subgroups, with statistical significance.

Comparison of the main clinicopathological variables between *BRAF*-mutated DTC, *RAS*-mutated, *TERT/TP53*-mutated DTC, all fusion-positive DTC, and WT cases showed no statistical difference in sex (*P* = 0.62); conversely, a statistical difference in median age was found (*P* = 0.0001). In particular, gene fusion positive DTCs were associated with younger patients. As expected, there was a statistical correlation between tumor histology and genetic alterations (*P* = 0.001). In particular, *BRAF* mutations were restricted to conventional PTC (100%), whereas *RAS* mutations were prevalent in FTC (77.8%) and OCC (11.1%) cases. One *PAX8-PPARG* rearranged case with solid/trabecular variant PTC histology had *RAS* comutations. Gene rearrangements were only found in PTC cases. There was a statistical correlation between the N-stage and genetic alterations (*P* = 0.0001): mutation-positive DTC and fusion-positive DTC were N-positive in 45.4% and 68.0% of cases, respectively.

### M-stage, radioactive iodide uptake/disease status, and time to radioactive iodide uptake refractory status according to genetic alterations

Assuming that *TERT/TP53* mutations, both as a single event or as comutations together with *BRAF* or *RAS* mutations, have a negative prognostic value, we have stratified cases into five molecular subgroups, namely, (i) *BRAF*-mutated DTC as a single event; (ii) *RAS*-mutated DTC as a single event, (iii) *TERT/TP53*-mutated DTC as a single event or as comutations together with other driver gene alterations (*BRAF* mutations, *RAS* mutations, or gene fusions); (iv) gene fusions as a single event; and (v) WT (absence of any of aforementioned alterations; Supplementary Table S3, available at https://doi.org/10.1016/j.esmoop.2023.102039).

**Figure 2:**
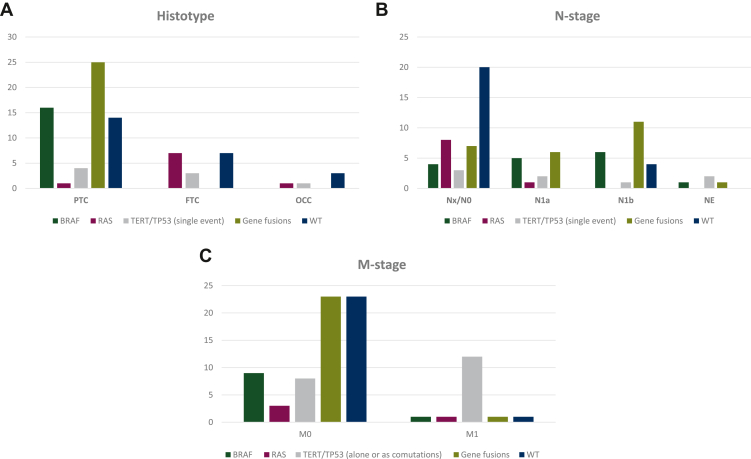
(A) Correlation between M-stage and genetic alterations; (B) correlation between radioactive iodide refractory (RAI-R) development and genetic alterations; (C) correlation between RAI uptake/disease status and genetic alterations. FTC, follicular thyroid carcinoma; OCC, oncocytic thyroid carcinoma of follicular cells; PTC, papillary thyroid carcinoma; WT, wild type.

As expected, there was a statistical correlation between M-stage and genetic alterations (*P* = 0.0001). In particular, 75% of metastatic cases at diagnosis were positive for *TERT/TP53* mutations, both as a single event and as comutations together with other driver alterations, whereas most localized cases were WT (34.8%) and fusion-positive (34.8%; Table 2; Figure 2A).

**Table 2:** M-stage, RAI resistance, and RAI uptake/disease status according to genetic alterations

	*BRAF* (single event)	*RAS* (single event)	*TERT/TP53* (alone or as comutations)	Gene fusions	Wild type	*P* value
**M-stage**						
M0	9 (13.6)	3 (4.5)	8 (12.1)	23 (34.8)	23 (34.8)	**0.0001**
M1	1 (6.3)	1 (6.3)	12 (75.0)	1 (6.3)	1 (6.3)	
**RAI-R**						
No	6 (14.0)	—	1 (2.3)	18 (41.9)	18 (41.9)	**0.0001**
Yes	4 (10.3)	4 (10.3)	19 (48.7)	6 (15.4)	6 (15.4)	
**RAI uptake/disease status**						
RAI+/D–	5 (14.3)	—	—	14 (40.0)	16 (45.7)	**0.0001**
RAI+/D+	1 (12.5)	—	1 (12.5)	4 (50.0)	2 (25.0)	
RAI–/D+	4 (21.1)	1 (5.3)	5 (26.3)	4 (21.1)	5 (26.3)	
RAI–/PD	—	3 (15.0)	14 (70.0)	2 (10.0)	1 (5.0)	

RAI+/D+, RAI uptake and disease persistence; RAI+/D–, RAI uptake and disease remission; RAI–/D+, RAI resistance and disease persistence; RAI–/PD, RAI resistance and progressive disease; RAI-R, radioactive iodide refractory.

To better define the clinical role of tumor genotyping in the clinical management of patients with DTC, we then evaluated the correlation between RAI uptake/disease status and genetic alterations. After a median follow-up period of 66 months (range 12-301 months), 39 cases (47.6%) became RAI-R. According to RAI uptake/disease status, 35 cases (42.7%) were defined as RAI sensitive with disease remission (RAI+/D–), 8 (9.8%) as RAI sensitive with disease persistence (RAI+/D+), 19 (23.2%) as RAI resistant with disease persistence (RAI-/D+), and 20 (24.4%) as RAI resistant with progressive disease (RAI–/PD; Table 2). Of the latter, 16 patients (19.5%) were treated with lenvatinib as first-line therapy, while 1 (1.2%) was treated with sorafenib as first-line therapy, and then with lenvatinib. Importantly, two patients (2.4%) were treated with sorafenib, lenvatinib, and then NTRK inhibitors, and one patient (1.2%) was treated with lenvatinib as first-line therapy and then with a RET inhibitor, according to the molecular profile of the patient’s tumor.

There was a significant correlation between RAI-R development and genetic alterations (*P* = 0.0001). Indeed, 48.7% of RAI-R cases were positive for *TERT/TP53* mutations as a single event or as comutations together with other driver gene alterations (*BRAF* mutations, *RAS* mutations, or gene fusions), while the great majority of RAI-sensitive cases carried gene fusions (41.9%) or were WT (41.9%; Table 2; Figure 2B). RAI uptake/disease status was statistically associated with specific genetic alteration subgroups (*P* = 0.0001). Indeed, of the 35 RAI+/D– cases, 5 (14.3%) were positive for *BRAF* p.V600E mutation, 14 (40.0%) were positive for gene rearrangements (9 *NTRK* and 5 *RET*), and 16 cases (45.7%) were WT. None of the RAI+/D– cases carried *TERT/TP53* mutations as a single event or as comutations with other driver gene alterations. Conversely, of the 20 RAI–/PD cases, 14 (70.0%) were positive for *TERT/TP53* mutations as a single event or as comutations, 3 cases (15.0%) had *RAS* mutations, 2 cases (10.0%) had gene fusions, and only 1 case was WT (4.8%; Table 2; Figure 2C).

**Figure 3:**
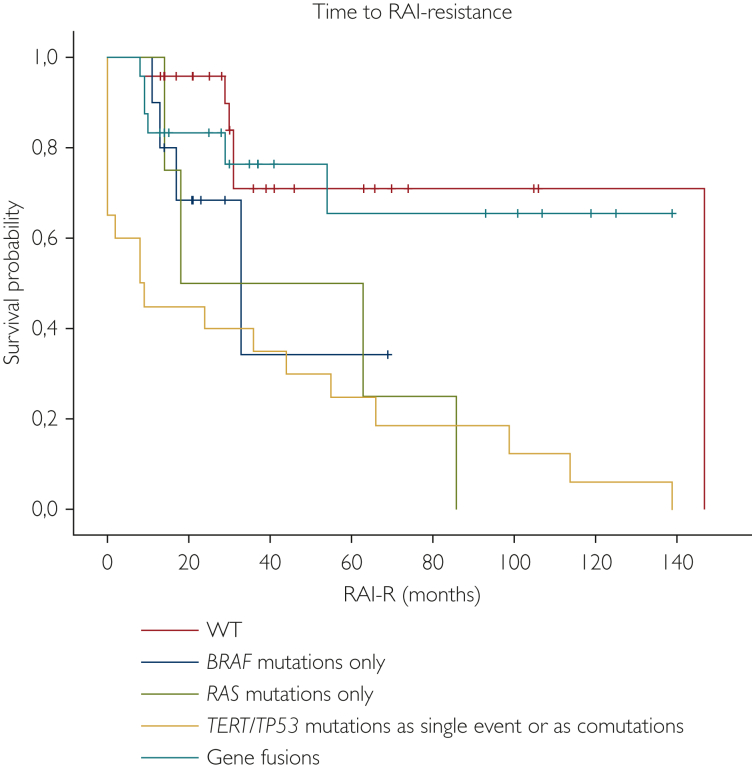
Time to RAI-resistance according to genetic alterations. RAI, radioactive iodide; RAI-R, radioactive iodide refractory; WT, wild type.

Not only RAI uptake/disease status, but also TTRR was significantly associated with genetic alterations (*P* = 0.0001). In particular, DTC with *TERT/TP53* mutations as a single event or as comutations displayed a shorter median TTRR of 35.4 months (range 15.0-55.8 months), compared with the median TTRR of fusion-positive DTC and WT DTC, respectively, which was 100.4 months (range 73.7-127.1 months) and 112.2 months (range 83.6-140.7 months; Supplementary Table S4, available at https://doi.org/10.1016/j.esmoop.2023.102039; Figure 3).

To assess whether the predictive value of genetic alterations for RAI-R development was independent of other known clinicopathological factors, univariate and multivariate Cox proportional hazard regression analyses were carried out (Supplementary Table S5, available at https://doi.org/10.1016/j.esmoop.2023.102039). By univariate analysis, T3-T4 stage [hazard ratio (HR) 4.45, 95% CI 2.09-9.47; *P* = 0.0001], *RAS* mutations only (HR 4.83, 95% CI 1.29-18.09; *P* = 0.01), and *TERT/TP53* mutations as a single event or as comutations (HR 5.98, 95% CI 2.22-16.13; *P* = 0.0001) were significantly associated with RAI-R development. T3-T4 stage (HR 3.82, 95% CI 1.69-8.63; *P* = 0.001) and *TERT/TP53* mutations as a single event or as comutations (HR 4.14, 95% CI 1.51-11.32; *P* = 0.006) remained independently associated with RAI-R after multivariate analysis.

## Discussion

In the past decades, molecular testing has played an ever-increasing role as an adjunct to preoperative fine-needle aspiration diagnosis of thyroid nodules, for risk stratification, and more recently as predictive of response to molecularly targeted therapy.^2^^,^^15^^,^^17^^,^^18^^,^^23^, ^24^, ^25^, ^26^, ^27^, ^28^ The principal genetic alterations of DTC are *NRAS*, *HRAS*, *KRAS*, *BRAF*, *PTEN*, *EIF1AX*, *DICER1*, *PIK3CA*, *TERT*, *TP53*, and *RET* mutations and *PAX8/PPARG*, *ALK*, *NTRK1*, and *NTRK3* rearrangements. They fall into two broad categories: driver alterations that promote tumor development, and secondary ones superimposed to the driver events promoting progression to high-grade tumors and anaplastic carcinoma. Among these secondary alterations are mutations of the *TERT* promoter, *PIK3CA*/*AKT*/*PTEN* pathway genes, and *TP53*.^1^^,^^3^^,^^26^^,^^29^^,^^30^ In particular, the coexistence of *BRAF* and *TERT* promoter mutations is associated with a subgroup of DTC with aggressive clinicopathological features, increased risk of distant metastasis, and poor outcomes.^9^^,^^11^^,^^25^^,^^31^, ^32^, ^33^, ^34^, ^35^ Thus, the inclusion of *TERT* promoter mutation analysis into routine clinicopathological evaluation is strongly recommended for accurate risk stratification.^26^^,^^36^ Mutations of *TP53* as well as those of *PIK3CA*/*AKT*/*PTEN* pathway genes represent additional secondary genetic markers of tumor aggressiveness. In fact, *TP53* alterations have been detected in 10%-35% of poorly differentiated thyroid tumors, and up to 80% in anaplastic thyroid carcinoma, while no *TP53* mutations are usually detected in conventional DTC.^26^

In the past years, molecular profiling has lately assumed even more substantial relevance, as *NTRK* and *RET* rearrangements have been recognized as actionable targets for metastatic RAI-R-DTC, inevitably expanding the spectrum of gene alterations to look for.^12^^,^^15^^,^^17^^,^^18^^,^^27^^,^^28^

Taking all these considerations together, molecular testing of DTC has now acquired great clinical relevance, because it not only allows to identify high-risk cases, but among these high-risk cases it also allows the identification of those with actionable genetic alterations. Thus it has become essential to tailor both patient active surveillance and systemic treatments. Nevertheless, to date, there remains no consensus on timing and algorithms to use for molecular testing in clinical practice.

In the present study, we report the results of molecular testing carried out on 82 patients with advanced DTC, investigating the clinical significance of molecular data, to highlight the relevance of tumor genotyping for the management of patients with DTC and to share a model for clinical practice.

Our NGS approach has allowed the identification of at least one DNA mutation in nearly half of the cases and of a gene rearrangement in approximately one-third of cases, confirming that DTC represents a heterogeneous group of tumors rather than one single entity, with distinct pathological features, molecular background, and clinical behavior.^1^ Our data thus confirm the existence of a strict correlation between tumor genotype, histologic phenotype, and clinicopathological features. Indeed, among mutation-positive DTCs, *BRAF* V600E mutation was confirmed to be almost restricted to conventional PTC, whereas *RAS* mutations prevalently belonged to FTC and OCC histotypes. Among fusion-positive DTCs, *NTRK1-3*, *RET,* and *ALK* rearrangements were limited to PTC and infiltrative follicular variant PTC histotypes.

First, in agreement with what has been already reported in the literature, we confirmed that *TERT* promoter, *TP53,* and *PIK3CA* mutations are associated with aggressive forms of DTC, in terms of both occurrence of distant metastases and earlier RAI-R onset.^11^^,^^32^, ^33^, ^34^, ^35^ Indeed, in our case series ∼50% of RAI-R cases were positive for *TERT/TP53* mutations as a single event or as comutations together with other driver gene alterations (*BRAF* mutations, *RAS* mutations, or gene fusions), and in this molecular subgroup, the TTRR was significantly shorter than in the other subgroups, confirming that *TERT* promoter comutations affect RAI avidity.^36^ Noteworthy, the presence of *TERT* promoter, *TP53,* and *PIK3CA* mutations, as both single events and comutations, was found to be significantly correlated with RAI-R development, independent of other known pathological parameters. Therefore, our data show that upfront tumor genotyping of advanced DTC should always include at least *TERT* and *TP53*, to identify those high-risk patients for whom systemic treatment could be considered earlier in case of persistent or recurrent disease.

Second, based on our data, molecular analysis of those genes commonly rearranged in DTC has led us to identify fusion events in approximately one-third of cases. As known, fusion-positive DTCs comprise a peculiar subset of patients, with specific clinicopathological features such as younger age of onset, PTC morphology, and the triad of multinodular growth, intratumoral fibrosis, and extensive lymphovascular invasion.^37^ In our series, fusion-positive DTC presented a median age at diagnosis of 41 years, with a slightly lower median age in cases with *NTRK1* and *NTRK3* fusions. N1a and N1b nodal extensions were detected in 24% and 44% of cases, respectively. Multifocality was found in more than half of our gene fusion cases. Overall, fusion-positive cases displayed a better prognosis. In the Cox proportional hazard regression analysis, the presence of gene rearrangements was not correlated with RAI-R development. However, six patients with gene fusion tumors became RAI-R within 20 months from the initial diagnosis. Two of them, both with *NTRK*-rearranged progressive disease, were treated with NTRK inhibitors. An additional case positive for *RET* rearrangement with *TERT* promoter and *TP53* comutations, and with advanced disease at presentation was treated with a RET inhibitor.^19^ Therefore, molecular testing including gene fusions should be routinely recommended for all patients with advanced DTC, because the early recognition of actionable genetic alterations allows to offer the appropriate molecularly driven treatment for these patients. However, gene fusions are more cumbersome (e.g. they often require higher tumor cell enrichment for reliable identification) and expensive to test compared with single-nucleotide variants and small insertion–deletions.^38^^,^^39^ Therefore because they are mutually exclusive with the other easier-to-detect driver alterations, it is very reasonable and cost-effective to first test all advanced DTC for SNP and small insertion–deletions, and then test the negative cases for gene fusions.

Although our findings may add important information on the clinical relevance of genetic testing in the management of patients with DTC, we acknowledge some study limitations. For sure, the retrospective design of the study, involving a subset of more aggressive DTC, may represent a great bias for interpreting the results. Thus a prospective validation on a larger case series is required to better define the prognostic role of molecular testing in DTC.

In conclusion, these data confirm the clinical relevance of genetic testing, both by comprehensive NGS and by a two-step approach, for all patients with advanced DTC, to identify both the subset of more aggressive tumors and the subset of tumors harboring actionable gene fusions. Therefore genetic testing should be included as part of the clinical workup and carried out as early as possible in all patients with advanced DTC, to ensure them the most appropriate management.
