# Corrigendum to “Clinical relevance of gene mutations and rearrangements in advanced differentiated thyroid cancer” [ESMO Open 8 (2023) 102039]

**DOI:** 10.1016/j.esmoop.2024.103704

**Published:** 2024-10-18

**Authors:** M. Nannini, A. Repaci, M.C. Nigro, A. Colapinto, V. Vicennati, T. Maloberti, E. Gruppioni, A. Altimari, E. Solaroli, E. Lodi Rizzini, F. Monari, A. De Leo, S. Damiani, U. Pagotto, M.A. Pantaleo, D. de Biase, G. Tallini

**Affiliations:** 1 Department of Medical and Surgical Sciences (DIMEC), University of Bologna, Bologna; 2 Medical Oncology, IRCCS Azienda Ospedaliero-Universitaria di Bologna, Bologna; 3 Division of Endocrinology and Diabetes Prevention and Care, IRCCS Azienda Ospedaliero-Universitaria di Bologna, Bologna; 4 Solid Tumor Molecular Pathology Laboratory, IRCCS Azienda Ospedaliero-Universitaria di Bologna, Bologna; 5 Endocrinology Unit-Azienda USL di Bologna, Bologna; 6 Division of Radiation Oncology, IRCCS Azienda Ospedaliero-Universitaria di Bologna, Sant’Orsola-Malpighi Hospital, Bologna; 7 Pathology Unit, Department of Pathology, Bellaria & Maggiore Hospital, AUSL di Bologna, Bologna; 8 Department of Pharmacy and Biotechnology (FaBiT), University of Bologna, Bologna, Italy

The authors regret that in the original publication Figure 2 was given incorrectly. The correct Figure 2 is given below.

**Figure:**
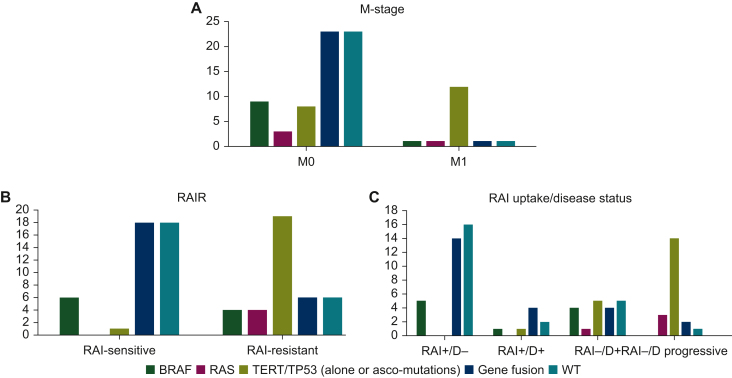

The authors would like to apologise for any inconvenience caused.

