# Supplementary material for: Clinical relevance of gene mutations and rearrangements in advanced differentiated thyroid cancer
Source: ESMO Open. 2023 Oct 23;8(6):102039. doi: 10.1016/j.esmoop.2023.102039 (PMC10774965; doi:10.1016/j.esmoop.2023.102039)
Supplement: Supplementary files [file mmc1.docx]

**Supplementary Figure 1**

**Suppl Figure 1**: conventional papillary carcinoma with high grade features and mitotic activity (Arrow in inset). The tumor has RET-CCDC6 fusion with TERT promoter, PIK3CA and TP53 co-mutations. The patient was successfully treated with the RET-inhibitor.

**Suppl Table 1: Clinic-pathological characteristics**

| **Total 82** | | |
| --- | --- | --- |
|  | **Median (range) yy** | **N (%)** |
| **Age** | 47 (20-86) |  |
| **Gender**  F  M |  | 54 (65.9)  28 (34.1) |
| **Histotype**  PTC  E-FV-PTC  FTC  OCC |  | 60 (73.2)  6 (7.3)  11 (13.4)  5 (6.1) |
| **T-stage**  T1  T2  T3  T4  NE |  | 26 (31.7)  16 (19.5)  29 (35.4)  7 (8.5)  4 (4.9) |
| **N-stage**  N0/Nx  N1a  N1b  NE |  | 42 (51.2)  14 (17.1)  22 (26.8)  4 (4.9) |
| **Multifocality**  No Yes  NE |  | 44 (53.7)  34 (41.5)  4 (4.9) |
| **Surgery**  No surgery HT TT  TT + central ND  TT + central/lateral ND |  | 4 (4.9)  5 (6.1)  38 (46.3)  14 (17.1)  21 (25.6) |
| **M-stage**  M0  M1 |  | 66 (80.5)  16 (19.5) |

PTC = papillary thyroid carcinoma; E-FV-PTC = encapsulated follicular variant papillary thyroid carcinoma; FTC = follicular thyroid carcinoma; OCC = oncocytic thyroid carcinoma of follicular cells; NE= not evaluable (no primary surgery); HT = hemithyroidectomy; TT = total thyroidectomy; ND = node dissection

**Suppl. Table 2: Genetic alterations identified**

|  |  | **N° (%)** |
| --- | --- | --- |
| **Point Mutations 33 (40.2)** | | |
| **BRAF**  **(19.5%)** | *BRAF* V600E | 10 (12.1) |
|  | *BRAF* V600E + *TERT* promoter | 5 (6) |
|  | *BRAF* V600E + *TERT* promoter + *TP53* | 1 (1.1) |
| **RAS**  **(11.0%)** | *HRAS* | 1 (1.1) |
|  | *NRAS* | 2 (2.4) |
|  | *NRAS* + *TERT* promoter | 1 (1.1) |
|  | *KRAS* + *TERT* promoter | 2 (2.4) |
|  | *HRAS* + *TERT* promoter | 1 (1.1) |
|  | *NRAS* + *AKT1* | 1 (1.1) |
|  | *HRAS* + *PAX8-PPARG* | 1 (1.1) |
| **Other**  **(9.7%)** | *TERT* promoter | 4 (4.8) |
|  | *TERT* promoter + *RNF43* | 1 (1.1) |
|  | *TERT* promoter + *PIK3CA* | 1 (1.1) |
|  | *TP53* | 2 (2.4) |
| **Rearrangements 25 (30.5)** | | |
| **NTRK**  **(17.1%)** | *NTRK3-ETV6* | 11 (13.4) |
|  | *NTRK1-TPM3* | 1 (1.1) |
|  | *NTRK3-EML4* | 1 (1.1) |
|  | NTRK1^*^ | 1 (1.1) |
| **RET** | *RET-CCDC6* | 6 (7.3) |
|  | *RET-NCOA4* | 2 (2.4) |
|  | *RET-CCDC6 + TERT* promoter + *PIK3CA* + *TP53* | 1 (1.1) |
| **ALK**  **(2.4%)** | *ALK-STRN* | 1 (1.1) |
|  | *ALK-EML4* | 1 (1.1) |

^*^Gene fusion identified by FISH, undefined rearrangement partner

**Suppl. Table 3: List of cases including into TERT/TP53 mutations, both as single event or as as co-mutations**

| **Type of mutations** | **N° (%)** |
| --- | --- |
| *BRAF* V600E + *TERT* promoter | 5 (6.0) |
| *BRAF* V600E + *TERT* promoter + *TP53* | 1 (1.1) |
| *NRAS* + *TERT* promoter | 1 (1.1) |
| *KRAS* + *TERT* promoter | 2 (2.4) |
| *HRAS* + *TERT* promoter | 1 (1.1) |
| *TERT* promoter | 4 (4.9) |
| *TERT* promoter + *RNF43* | 1 (1.1) |
| *TERT* promoter + *PIK3CA* | 1 (1.1) |
| *TP53* | 2 (2.4) |
| *RET-CCDC6 + TERT* promoter + *PIK3CA* + *TP53* | 1 (1.1) |

**Suppl. Table 4: Time to RAI-resistance according to the molecular status**

|  | |  |  | |  |  |
| --- | --- | --- | --- | --- | --- | --- |
|  | ***BRAF***  **(single event)** | ***RAS***  **(single event)** | ***TERT/TP53***  **(alone or as co-mutations)** | **Gene fusions** | **WT** | **P value** |
| **N° event (%)** | 4 (40.0) | 4 (100.0) | 19 (95.0) | 6 (25.0) | 6 (25.0) | **<0.0001** |
| **N° Truncated (%)** | 6 (60.0) | - | 1 (5.0) | 18.0 (75.0) | 18 (75.0) |  |
| **Median TTRR (months)** | 39.3 | 45.2 | 35.4 | 100.4 | 112.2 |  |
| **Std error** | 10.4 | 17.5 | 10.4 | 13.6 | 14.6 |  |
| **Range (95% CI)** | 18.9-59.6 | 10.8-79.6 | 15.0-55.8 | 73.7-127.1 | 83.6-140.7 |  |

TTRR= Time to RAI-resistance

**Suppl. Table 5: Univariate and multivariate Cox proportional hazard regression analyses of predictive factors for RAI-R development**

| **RAIR** | **Univariate Cox Regression** | | **Multivariate Cox Regression** | |
| --- | --- | --- | --- | --- |
|  | **HR (95%CI)** | ***p-value*** | **HR (95%CI)** | ***p-value*** |
| **Histotype**  PTC  E-FV-PTC  FTC  OCC | 1  5.06 (0.96-26.63)  0.84 (0.22-3.09)  1.33 (0.34-5.11) | ns  ns  ns | ns | ns |
| **T-stage**  T1-T2  T3-T4 | 1  4.45 (2.09-9.47) | **0.0001** | 3.82 (1.69-8.63) | **0.001** |
| **N- stage**  N0/Nx  N1a-N1b | 1  1.38 (0.72-2.61) | ns | ns | ns |
| **Multifocality**  No  Yes | 1  0.95 (0.50-1.82) | ns | ns | ns |
| **Tumor genotyping**   - WT - *BRAF* mutations only - *RAS* mutations only - *TERT/TP53* mutations alone or as co-mutations - Gene fusions | 1  2.80 (0.74-10.55)  4.83 (1.29-18.09)  5.98 (2.22-16.13)  1.24 (0.37-4.08) | ns  **0.01**  **0.0001**  ns | 3.48 (0.91-13.35)  3.02 (0.78-11.58)  4.14 (1.51-11.32)  0.99 (0.29-3.34) | ns  ns  **0.006**  ns |

PTC: papillary thyroid carcinoma; E-FV-PTC = encapsulated follicular variant papillary thyroid carcinoma; FTC: follicular thyroid carcinoma; OCC: oncocytic thyroid carcinoma of follicular cells
